# Pond Assay for the Sensory Systems of *Caenorhabditis elegans*: A Novel Anesthesia-Free Method Enabling Detection of Responses to Extremely Low Chemical Concentrations

**DOI:** 10.3390/biology11020335

**Published:** 2022-02-21

**Authors:** Michiyo Suzuki, Yuya Hattori, Toshiyuki Saito, Yoshinobu Harada

**Affiliations:** 1 Takasaki Advanced Radiation Research Institute, National Institutes for Quantum Science and Technology (QST-Takasaki), 1233 Watanuki, Takasaki 370-1292, Gunma, Japan; y-hattori@kure-nct.ac.jp; 2 National Institute of Radiological Sciences, National Institutes for Quantum Science and Technology (QST-NIRS), 4-9-1 Anagawa, Inage-ku, Chiba 263-8555, Chiba, Japan; saito.toshiyuki@qst.go.jp; 3 Human Resources Development Center, National Institutes for Quantum Science and Technology (QST-CHRD), 4-9-1 Anagawa, Inage-ku, Chiba 263-8555, Chiba, Japan; harada.yoshinobu@qst.go.jp

**Keywords:** pond assay, chemotaxis, response detection, experimental accuracy, *Caenorhabditis elegans*

## Abstract

**Simple Summary:**

We propose a pond assay for the sensory systems (PASS) of *Caenorhabditis elegans* as a novel method of behavioral analysis. In PASS, the test solution is injected into a recess(es) formed on agar and the response of *C. elegans* to its odor and/or taste is examined. Once *C. elegans* individuals fall into recesses (ponds) filled with liquid, they cannot return to the solid medium. In this way, the animals are trapped with certainty without the use of anesthesia. The anesthesia used to keep animals in the attractant area in conventional chemotaxis assays is no longer required, allowing pure evaluation of the response to specific substances. Furthermore, the test itself can be greatly streamlined because the preparation can be completed simply by providing a recess(es) and filling the liquid. The present paper reports the detailed method and effectiveness of the novel PASS through a series of chemotaxis assays. By using the PASS method, we found that the olfactory system of *C. elegans* accurately senses odors even at extremely low concentrations lower than the previously known detection threshold. This method can be applied to biosensor technology that uses *C. elegans* to detect chemical substances present at extremely low concentrations in environmental samples and biological samples with high sensitivity.

**Abstract:**

Chemotaxis in the nematode *Caenorhabditis elegans* has basically been examined using conventional assay methods. Although these can be problematic, for example, in their use of anesthesia, the method has never been improved. We propose a pond assay for the sensory systems (PASS) of *C. elegans* as a novel population-based method of behavioral analysis. The test solution is injected into a recess(es) formed on agar and the response of *C. elegans* to its odor and/or taste is examined. Once *C. elegans* individuals fall into recesses (ponds) filled with liquid, they cannot return to a solid medium. In this way, the animals are trapped with certainty without the use of anesthesia. The anesthesia used to keep animals in the attractant area in conventional chemotaxis assays is no longer required, allowing pure evaluation of the attractant or repellent response to specific substances. Furthermore, the assay itself can be greatly streamlined because the preparation can be completed simply by providing a recess(es) and filling the liquid. The present paper reports the detailed method and effectiveness of the novel PASS.

## 1. Introduction

The nematode *Caenorhabditis elegans* is a well-established model organism for investigating vital mechanisms, such as neural behaviors, development, and aging [1,2]. The animal shows sensory responses, so-called “taxis”, to specific chemicals (chemotaxis) [3,4,5,6,7,8,9,10,11,12,13,14,15,16,17,18,19,20,21,22,23,24,25,26] and/or temperatures (thermotaxis) [11,12] and its associative learning [27,28,29,30]. In a chemotaxis assay, a concentration gradient of the test substance is formed on one side of an agar plate. To test chemotaxis with substances with an odor, the test solution is dropped on one side and the standard solution is spotted onto the other side as a control. To prevent the animals from moving out of the attractant area over time, an anesthesia, such as sodium azide, is added dropwise to both sides of the plate and the mixtures are then dried. However, it takes time to dry the test solution and the anesthesia, and the diffusion of the anesthesia over time affects the evaluation of the response to the substance being tested. In particular, when the animals enter the area where the anesthesia has been dropped, they cannot escape and are then trapped. This phenomenon makes an accurate response evaluation difficult and the factual nature of the conclusions cannot be guaranteed. Furthermore, animals are often affected by adaptation to chemical substances and associative learning over time, thus there is an additional problem that the results can vary from trial to trial due to the effects of anesthesia and adaptation.

The experimental error between assays in biological experiments has been explained as being due to “individual differences in the living organisms tested”. The chemotaxis assay of *C. elegans* has been established for over half a century, but the fact that the experimental results differ from laboratory to laboratory and that the experimental error is large have also been dismissed as being due to “individual differences in the living organisms”. However, we consider that, in most *C. elegans* assays, the error between assays does not actually depend on individual differences in *C. elegans*, but rather on the assay method, the proficiency of the researchers, and/or the experimental environment. Regardless of these serious problems, the conventional assay method for the sensory systems of *C. elegans* has not been greatly improved for a long time. If it is known that error is likely to occur between assays depending on the skill level of the experimenters, it is important to improve the assay method so that such error is less likely to occur.

In the present study, we propose a pond assay for the sensory systems (PASS) of *C. elegans* as a powerful and effective novel method of behavioral assay.

## 2. Materials and Methods

### 2.1. Strains and Culture

*Caenorhabditis elegans* hermaphrodites of the wild-type strain N2 were grown at 20 °C on a 10-cm Petri dish (IWAKI 100 mm/non-treated dish; AGC Techno Glass Co., Ltd., Shizuoka, Japan) containing 20 mL of nematode growth medium (NGM) [1]. *Escherichia coli* OP 50 strain (food source) was spread on the NGM plate and incubated overnight in advance. The developmental stages of *C. elegans* were synchronized from the embryo stage, and well-fed young adults were used.

### 2.2. Basics of Preparation of a Novel Plate for the Pond Assay for the Sensory Systems (PASS Plate)

We developed a novel plate for the pond assay for the sensory systems (PASS plate). The PASS plate for trapping *C. elegans* comprises a solid phase. Agar medium (5 mL of 1 M potassium phosphate (pH 6.0), 1 mL of 1 M CaCl_2_, 1 mL of 1 M MgSO_4_, and 20 g agar in 1 L of H_2_O; sterilized by autoclaving) [27] was used for a solid phase. To avoid the influence of variation in the degree of dryness (water content) of the solid medium, the assay plates with agar medium used for PASS and conventional assays were always produced on the day before the chemotaxis assays. Approximately 50 plates filled with agar medium were stored together in a plastic food storage container at room temperature.

**Figure 1:**
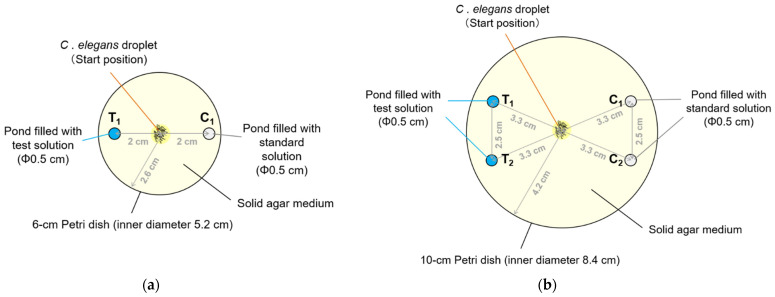
Schematic of PASS plates described in Section 2.2. (**a**) A ⌀6-cm PASS plate with two ponds, T_1_ for test solution and C_1_ for standard (control) solution. (**b**) A ⌀10-cm PASS plate with four ponds, T_1_ and T_2_ for test solution and C_1_ and C_2_ for standard solution.

**Figure 2:**
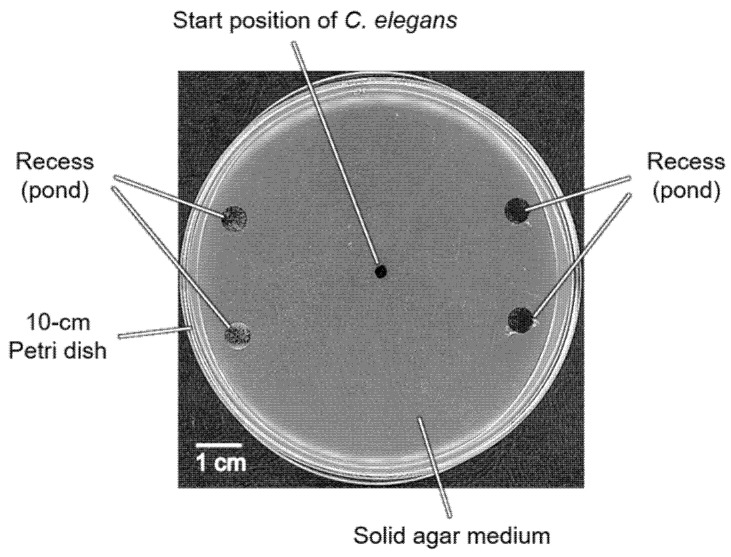
An overhead image of a ⌀10-cm PASS plate used for PASS to evaluate the chemotaxis of *C. elegans*.

On the day of the PASS assay, recesses for ponds of PASS plates were formed immediately before the chemotaxis assay. In the present study, the shape of recess was circular and the number of recesses was two or four depending on the assay conditions, as shown in Table 1. Figure 1a shows an overhead view of a PASS plate with two recesses of 0.5 cm in diameter, in which a 6-cm Petri dish was used. Furthermore, Figure 1b shows an overhead view of a PASS plate with four recesses of 0.5 cm in diameter, in which a 6-cm or 10-cm Petri dish was used as a container. The recess was formed so as to extend from the surface of the solid medium toward the bottom by hollowing out the solid medium (Figure 2) using a ⌀0.5 cm cylindrical cutting knife (Decorative knife: round, kai corporation. and kai industries Co., Ltd., Tokyo, Japan). In this procedure, a piece of paper with the layout of recesses (shown in Figure 1a,b) printed on it was placed under a plate and recesses were produced. A line was drawn on the edge of the Petri dish on the test side, so that the side to be used for testing can be easily recognized. As a more accessible fabrication tool, the use of biopsy punches that can produce recesses of different diameters can also be considered. Additionally, as another method to produce the PASS plate, we can consider forming recess(es) at the same time as formation of the solid phase, in which we can use a mold comprising at least one protrusion corresponding to at least one recess.

A mold that can be fixed to the Petri dish (container) is better for producing the PASS plate. 

Furthermore, a 6-cm plate and a 10-cm plate (as shown in Figure S1a,b) for the conventional assays were employed for comparative purposes.

### 2.3. Preparation of C. elegans Individuals for the PASS

The method of preparation of *C. elegans* before the assay followed the previously reported steps. The series of procedures here was basically started after filling recesses of the PASS plate with the test solution or standard (control) solution, and was completed within approximately 10 min. Briefly, after culturing, the wash buffer solution (5 mL of 1 M potassium phosphate (pH 6.0), 1 mL of 1 M CaCl_2_, 1 mL of 1 M MgSO_4_, and 0.5 g gelatin in 1 L of H_2_O; sterilized by autoclaving) [27] was injected into the culture plates of *C. elegans* and the liquid containing animals was collected by inhalation with a pipette and transferred to a centrifuge tube. After several minutes, the upper layer of the buffer solution containing impurities such as *E. coli* was removed and a new buffer solution was added to wash the animals. The upper layer was removed and the process repeated once more. By performing this step twice, the contaminants other than animals were reduced. In all assays described below, a droplet containing washed animals was supplied by spotting onto the center of the plate; the liquid was then wiped off using a paper wipe (Kimwipe S-200, NIPPON PAPER CRECIA Co., Ltd., Tokyo, Japan). In this way, *C. elegans* individuals started to move on the plate and the assay was initiated.

### 2.4. Counting the Number of C. elegans Individuals and Its Support Software

**Figure 3:**
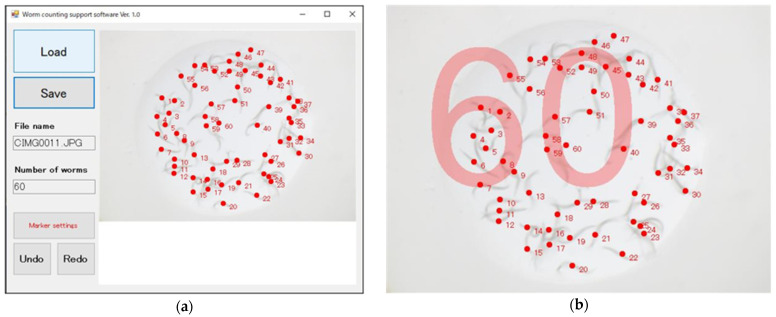
Overview of software to facilitate worm counting. (**a**) The screen of this software after counting has been completed. In the manual counting with this software, we load an image of *C. elegans* individuals trapped in a pond. A red circle as a marker and a serial number are presented by clicking on each body. (**b**) An image after counting all animals trapped in a pond. The total number of animals, 60 in this example, is presented.

When the predetermined duration for the assay had passed, an image of each pond was captured by a digital camera mounted on a microscope. Animals in the image were counted manually. For easy and rapid counting, we developed and used a software that supports manual counting of the animals (Figure 3a, Software S1). In this software, we load an image of *C. elegans* individuals trapped in a pond, click the body of each animal, after which a circle as a marker and a serial number are presented. Upon completion of the counting, the save button is clicked and the total number of animals is written in the image (Figure 3b). For counting the number of animals, the use of corresponding Fiji Plugins such as that based on Cell Counter can also be considered to prevent human error, especially in large-scale studies.

### 2.5. Selection of Liquid Appropriate for a Standard Solution of the PASS

To evaluate the effects of liquid used for both a standard solution and a solvent of the substance tested, we investigated the preference of *C. elegans* among the following three liquids used for PASS: ultrapure water (Milli-Q Plus, Merck, France; sterilized by autoclaving), wash buffer solution, and physiological saline (Isotonic Sodium Chloride Solution CMX; Nichi-Iko Pharmaceutical Co., Ltd., Toyama, Japan). We used a 6-cm PASS plate containing 10 mL of solid agar medium with two recesses, as shown in Figure 1a. We performed comparisons of all pairs of these three liquids. From at least 1 h before the chemotaxis assay until its end, the laboratory temperature and materials used were kept at 20 °C. For each pair, assays were performed three times independently. Each recess was filled with approximately 100 µL of a liquid to the brim, and a droplet containing approximately 100 washed animals was spotted on the center of a PASS plate and released by wiping off the liquid from the droplet. A lid was put on the plate and the plate was sealed with paraffin film (Parafilm; Bemis, IL, USA). Finally, the entire plate was covered with a light-shielding cover and allowed to stand for at least 30 min. After 30 min had elapsed, an image of each pond was obtained, and the number of animals was counted. The percentage of animals trapped in each pond was calculated and the preference was compared between two liquids based on the mean values of three independent assays. 

### 2.6. PASS for Detection of Olfactory Response of C. elegans to Diacetyl 

From at least 1 h before the chemotaxis assay until its end, the laboratory temperature and materials used were kept at 20 °C. The volume of the agar medium (thickness of solid phase) and the liquid used as standard (control) liquid and solvent differed between the assays. The conditions of each assay below (conditions 1–5) are shown in Table 1. Undiluted diacetyl was diluted from 10^−1^ to 10^−6^ by the predetermined liquid (wash buffer solution or ultrapure water) immediately before the assay. Procedures for the chemotaxis assay of diacetyl using the novel PASS method and the conventional method are described below. 

#### 2.6.1. PASS 

Besides the use of the novel PASS plate, chemotaxis assays were performed by previously reported methods [9,24], with some modifications, to evaluate the chemotaxis of *C. elegans* to diacetyl. For the PASS plates used under conditions 1–4, two points 3.3 cm away from the center of the 10-cm plate and the diametrically opposite points were excised as recesses for ponds, as shown in Figure 1b. We decided on the positions in the pond in the case of the 10-cm plate based on the previous method as shown in Figure 1b. In addition, for PASS plates used under conditions 5, a point 2 cm away from the center of the 6-cm plate and the diametrically opposite point were excised as recesses for ponds, as shown in Figure 1a. A predetermined volume of a graded series of dilutions or undiluted diacetyl solution was injected into the pond(s) on the test side. Subsequently, the same volume of standard solution was injected into the pond(s) on the control side. A droplet containing between 100 and 200 washed animals was spotted on the center of each plate and released by wiping off the liquid from the droplet. A lid was put on each plate and the plate was sealed with paraffin film. To prevent odor transfer, each plate was placed at a sufficient distance away from each other and left to stand. Finally, the entire plate was covered with a light-shielding cover and left to stand for 1 h. An image of each pond was obtained. Based on these images, the number of animals was counted and the chemotaxis index (C.I.) was calculated as follows: C.I. for the PASS = (the number of animals trapped in the pond(s) on the test side − the number of animals trapped in the pond(s) on the control side)/(the number of animals trapped in all ponds). 

#### 2.6.2. Conventional Assay

One microliter of each of a graded series of dilutions (10^−6^, 10^−5^, 10^−4^, 10^−3^, 10^−2^, 10^−1^) or undiluted diacetyl solution was spotted on two points, T_1_ and T_2_, at one end of the assay plates, and the same volume of standard solution was spotted on two points, C_1_ or C_2_, at the control side, as shown in Figure S1b. Subsequently, 1 μL of 0.5 M sodium azide was spotted on each point, T_1_, T_2_, C_1_, or C_2_, at both ends of the plates to trap the animals moving in the area, based on a previously reported method [9,24]. The subsequent procedure was basically the same as that for PASS. After 1 h had elapsed, an image of the whole plate was obtained using an image scanner. Using this image, the number of animals was counted and chemotaxis index (C.I.) was calculated as follows: C.I. for the conventional method = (the number of animals in the test area shown in a dotted line in Figure S1b − the number of animals in the control area shown in a dotted line in Figure S1b)/(the number of animals in the test area + the number of animals in the control area).

### 2.7. PASS for Detection of Taste Response of C. elegans to NaCl

From at least 1 h before the chemotaxis assay until its end, the laboratory temperature and materials used were kept at 20 °C. One day before the chemotaxis assay, a 100 mM NaCl agar plug was excised from a 10-cm plate containing 20 mL of NaCl agar medium (100 mL of 1 M NaCl, 5 mL of 1 M potassium phosphate (pH 6.0), 1 mL of 1 M CaCl_2_, 1 mL of 1 M MgSO_4_, and 20 g of agar in 1 L of H_2_O; sterilized by autoclaving) using a ⌀0.5-cm cylindrical cutting knife. Then, a ⌀0.5-cm NaCl plug was placed on the surface of the 6-cm plate containing 3 mL of agar medium described in Section 2.2. The NaCl plug was left overnight (approximately 16–18 h) and was removed shortly before the chemotaxis assay. In this way, the concentration gradient of 100 mM NaCl was formed. Procedures for chemotaxis assay of NaCl using the novel PASS method and the conventional method are described below. 

#### 2.7.1. PASS

Besides the use of the novel PASS plate, chemotaxis assays were performed by following previously reported methods [6,27,30] to evaluate the chemotaxis of *C. elegans* to sodium chloride (NaCl) with some modifications. As shown in Figure 1a, the point, T_1_, where a NaCl plug was initially placed was excised as a recess for a pond. In addition, as a control, a recess was formed at a position, C_1_, approximately 4 cm away from the center of the point where a NaCl plug was placed. Each recess was filled with approximately 25 µL of a wash buffer solution at 20 °C. A droplet containing approximately 100 washed animals was spotted on the center of each plate and released by wiping off the liquid. A lid was put on each plate and the plate was sealed. Finally, the entire plate was covered with a light-shielding cover and left to stand for 30 min. After 30 min had elapsed, images of the whole plate or ponds were obtained, and the number of animals was counted. Chemotaxis index was basically calculated by the same equations as for the above olfactory assays. 

#### 2.7.2. Conventional Assay

The 6-cm assay plates with a concentration gradient of 100 mM NaCl were formed as described above. One microliter of 0.5 M sodium azide was spotted on the center of the NaCl concentration gradient, T_1_, and that of the control spot, C_1_, as shown in Figure S1a to trap the animals moving in the area based on a previously reported method. The subsequent procedure of chemotaxis assay of NaCl was performed by following the above method for PASS. After 30 min had elapsed, an image of the whole plate was obtained, and the number of animals within the area shown in a dotted line in Figure S1a was counted. The chemotaxis index was basically calculated by the same equations as for the above olfactory assays.

### 2.8. Statistical Analysis

Statistical analysis was performed on data from more than three independent trials of the chemotaxis assay. The significance of differences between each of the concentration and control groups was analyzed by one-way analysis of variance (ANOVA), except for the chemotaxis assay to NaCl. *p* < 0.001, *p* < 0.01, or *p* < 0.05 was considered significant. Note that the data with no variance were excluded from the analysis. In the chemotaxis assay to NaCl, after confirming the homoscedasticity using F-test, the significance of differences between the conventional method and PASS method was examined using Student’s *t*-test. Here, *p* < 0.05 was considered significant. Numerical values are presented as values with standard errors of the mean (SEM). All statistical analyses were performed using Microsoft Excel software (Microsoft, Redmond, WA, USA).

## 3. Results

### 3.1. Development of Pond Assay for the Sensory Systems of C. elegans

We proposed a pond assay for the sensory systems, PASS, applicable for detecting the chemotaxis of *C. elegans*. In this assay, multiple ponds were produced on an agar medium and filled with a test solution or standard (control) solution. Figure 2 shows an overhead view of a PASS plate with four ponds. In this example, as shown in Figure 1b, two recesses (T_1_ and T_2_) on the left side were filled with test solution, and two recesses (C_1_ and C_2_) on the right side were filled with standard solution, and almost all animals were trapped in the left ponds. To count the number of animals in the ponds, we originally developed “the worm counting support software” (Figure 3a, Software S1). After loading an image of a pond, we selected each animal by clicking it, and then the serial number was presented. Finally, by saving the image, the total number of animals counted was fixed and presented as shown in Figure 3b. This software enabled easy and accurate counting of the animals. In the subsequent section, we present the results of the assay based on this PASS method. 

### 3.2. Selection of Liquid Appropriate for a Standard Solution Filling the Ponds of the PASS

To evaluate the effects of a liquid used for both standard solution and solvent of the substance tested, we investigated the preference of *C. elegans* between pairs of liquids among the following candidates: ultrapure water, wash buffer solution, and physiological saline. 

**Figure 4:**
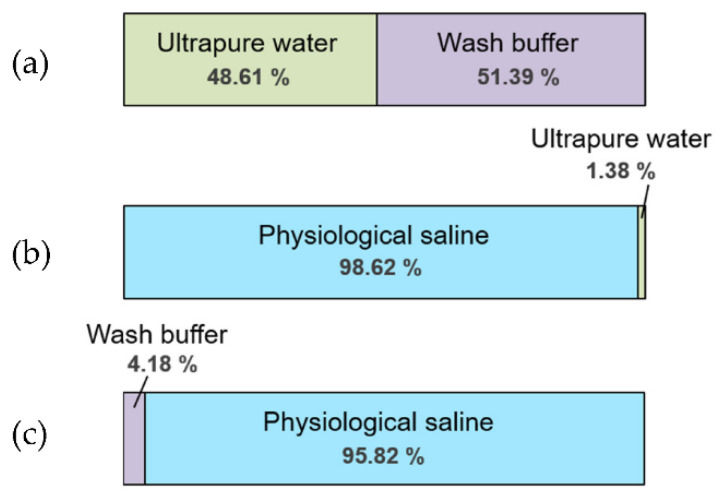
Result of PASS of *C. elegans* for liquid selection. (**a**) Result of selection between ultrapure water and wash buffer. (**b**) Result of selection between physiological saline and ultrapure water. (**c**) Result of selection between wash buffer and physiological saline. Green bar, light-purple bar, and light-blue bar represent the proportions of animals trapped in ponds filled with ultrapure water, wash buffer solution, and physiological saline, respectively. PASS was performed three times independently for each pair of liquid, from which the average percentage of each assay was obtained.

The proportions of animals trapped in a pond filled with ultrapure water and in a pond filled with wash buffer solution were almost the same (Figure 4a). Overall, 98.62% of animals were trapped in the pond filled with physiological saline, while only 1.38% were trapped in the pond filled with ultrapure water (Figure 4b). Furthermore, 95.82% of animals were trapped in the pond filled with physiological saline and only 4.18% were trapped in the pond filled with wash buffer solution (Figure 4c). The fact that the animals approached physiological saline indicates that they preferred it or were repelled by ultrapure water or wash buffer solution. Considering that *C. elegans* prefers NaCl at a concentration around 100 mM [27], it was possible that the salt in the physiological saline diffused into the solid medium around the pond and the animals were induced to approach it by its taste. Animals clearly tended to approach physiological saline. Therefore, to prevent effects of taste on the chemotaxis assay of an odorant(s), tasteless and odorless solvents such as ultrapure water and wash buffer solution are appropriate when testing by dissolving a substance(s). Even if the tasteless solution cannot be selected, it is required that a standard solution (buffer) for control should be used as a solvent for test solution. In contrast, it was considered that physiological saline should be used as a control when analyzing samples such as human blood containing salt at almost the same dose. Furthermore, from this result, it can be confirmed that the chemotaxis assay of taste can be conducted by filling taste solution in the recessof a PASS plate. 

### 3.3. Detection of Response of C. elegans to Diacetyl Based on the PASS

To evaluate the effectiveness of the PASS for detecting olfactory chemotaxis, we selected diacetyl, which is a single odorant for which the response of *C. elegans* has been well investigated. It was previously reported that a pair of ASH sensory neurons responded to high diacetyl concentrations only, whereas a pair of AWA sensory neurons reacted to both low and high concentrations [23,24,25]. We performed PASS to detect the response of *C. elegans* to 6- or 7-step dilutions (1 to 1 × 10^−5^ or 1 × 10^−6^) of diacetyl under variable conditions, 1 to 5, as shown in Table 1. It was shown that *C. elegans* escapes from high concentrations of diacetyl and prefers low concentrations of it [25]. Through a series of assays described below, we evaluated the effects of factors, such as type of the method, volume of agar medium, type of solvent, size of the plate, and the number of assay iterations, on the result of assay.

#### 3.3.1. Comparison of the Conventional Method and the Novel PASS Method

To evaluate the basic performance of the PASS, we performed three independent assays based on the conventional method of chemotaxis assay under condition 1 in Table 1, and three trials based on the PASS method under condition 2 in Table 1. We employed wash buffer solution as a solvent and a standard solution. 

**Figure 5:**
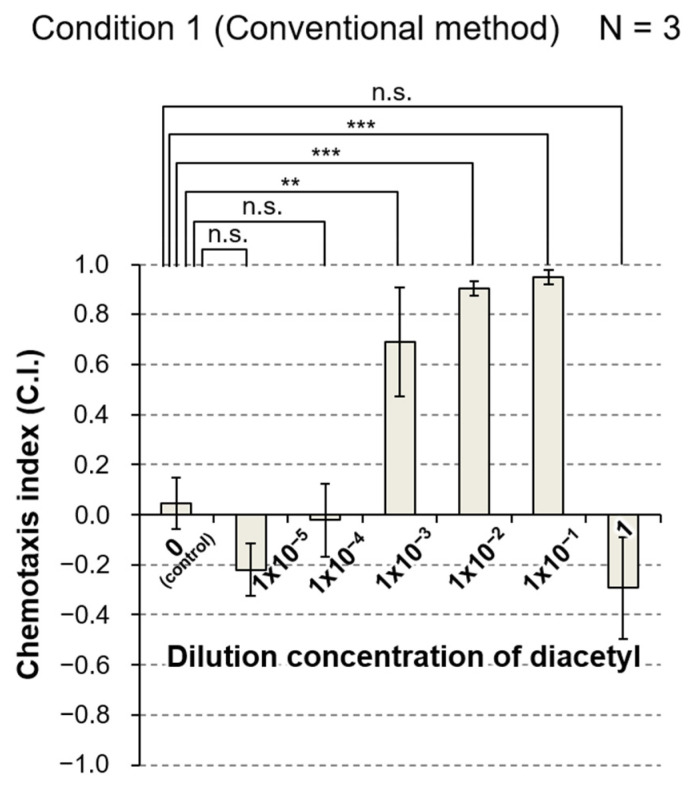
Results of conventional assay to evaluate chemotaxis of *C. elegans* to a graded series of concentrations of diacetyl. Three independent assays were performed under condition 1 in Table 1. The maximum concentration of diacetyl was 1 (undiluted solution) and the minimum diluted concentration was 1 × 10^−5^. Light- charcoal bar and error bar represent the mean and SEM of the chemotaxis index (C.I.) of three assays under the same condition. C.I. = (the number of animals in the test area − the number of animals in the control area)/(the number of animals in the test area + the number of animals in the control area). All data were analyzed by one-way ANOVA at *p* < 0.05, *p* < 0.01 (**), and *p* < 0.001 (***) significance levels by comparison to the control group. n.s., not significant; N, the number of assays. The details of the *p*-values are shown in Table S1.

As shown in Figure 5, in the chemotaxis assay based on the conventional method, animals approached the areas with each of three diluted concentrations, 10^−3^, 10^−2^, and 10^−1^, whereas they did not approach nor escape from the area spotted with diacetyl at the other concentrations (see Table S1 for *p*-values). In addition, large experimental errors were obtained, except for in the control, and at dilutions of 10^−2^ and 10^−1^. Although data indicating that animals approached diacetyl at a dilution of 10^−6^ have been reported by experts in chemotaxis assays [7,14,19], this tendency was not observed in three independent assays based on the conventional method, the same as in many other reports. This suggests that the reliable concentration range of detection of the conventional chemotaxis assays (N ≤ 3) performed by experimenters who are not experts in chemotaxis assays was from 10^−3^ to 1, but it does not indicate that this range of detection for diacetyl corresponds to the performance of the sensory systems of *C. elegans* itself. 

**Figure 6:**
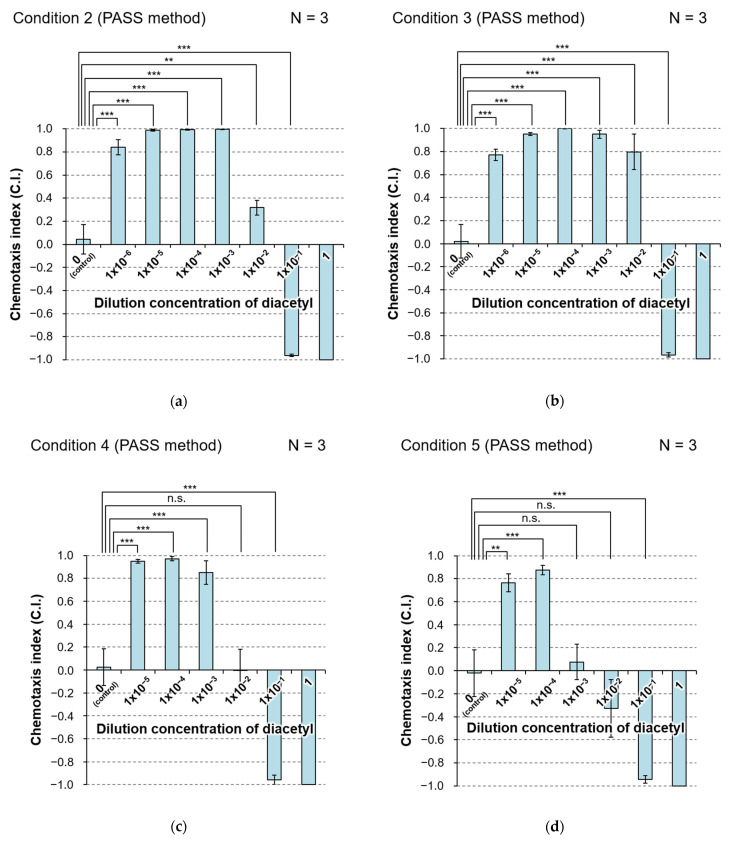
Result of PASS to evaluate chemotaxis of *C. elegans* to a graded series of concentrations of diacetyl. (**a**) Result of PASS under condition 2 in Table 1. (**b**) Result of PASS under condition 3 in Table 1. (**c**) Result of PASS under condition 4 in Table 1. (**d**) Result of PASS under condition 5 in Table 1. The maximum concentration of diacetyl was 1 (undiluted solution) and the minimum diluted concentration was 1 × 10^−6^ for (**a**,**b**), or 1 × 10^−5^ for (**c**,**d**). Light-blue bar and error bar represent the mean and SEM of chemotaxis index (C.I.) of three independent assays under the same condition. C.I. = (the number of animals trapped in allponds on the test side − the number of animals trapped in all ponds on the control side)/(the number of animals trapped in all ponds). All data were analyzed by one-way ANOVA at *p* < 0.05, *p* < 0.01 (**), and *p* < 0.001 (***) significance levels by comparison to the control group. Note that the data of the undiluted solution with no variance were excluded from the analysis. n.s., not significant; N, the number of assays. The details of the *p*-values are shown in Table S1.

In contrast, in the PASS, C.I. values of five dilutions from 10^−6^ to 10^−2^ were positive, whereas those of both 10^−1^ dilution and the undiluted solution were negative (Figure 6a; see Table S1 for *p*-values). This indicates that animals were trapped in the recesses filled with each of five dilutions from 10^−6^ to 10^−2^, whereas they were not trapped in the recesses filled with solution at 10^−1^ dilution and the undiluted solution. Relatively large experimental error was observed only for the result at 10^−2^ dilution, in which the C.I. changed to a positive value from a negative value, i.e., the response changed from avoidance to preference. From this result of the PASS, the errors of the sensory system depending on *C. elegans* individuals themselves may be small and within the range of one order of magnitude of dilution.

Since even in chemotaxis assays using test solutions with the same diluted concentration, the spatial concentration in the conventional method may be higher than that in the PASS method, the results of both cannot be compared with each other simply. However, the concentration range within which the PASS method performed reliable detection appeared to be from 1 to at least 10^−6^, indicating that *C. elegans* has olfactory sensors with the ability to detect odorants at concentrations covering this range.

#### 3.3.2. Effect of Volume of Agar Medium on Performance of PASS

To evaluate the effect of the volume (thickness) of agar medium corresponding to the pond depth, we employed agar medium twice as thick as in condition 2. We performed three independent assays based on the PASS method under this condition (condition 3 in Table 1). 

As shown in Figure 6b, the results showed the same tendency as for the PASS in Section 3.3.1, but the value of C.I. at 10^−2^ dilution was higher than that in condition 2 (see Table S1 for *p*-values). As a possible explanation of this, it was considered that the humidity in the PASS plate increased and the diffusion of the diacetyl solution from the pond was suppressed when the amount of agar medium was doubled. As a result, in condition 3, the concentration of diacetyl in air was generally lower than that in condition 2, and the population of animals that preferred the 10^−2^ diluent was increased. If it is true that *C. elegans* can detect such slight differences of diacetyl concentration, its olfactory sensor system might be much more sensitive than we suspected. 

#### 3.3.3. Effect of Type of Solvent on Performance of the PASS

To evaluate the effect of the type of solvent on the performance of the PASS, we employed ultrapure water as a solvent and standard solution instead of wash buffer solution in condition 2. We performed three independent assays based on the PASS method under this condition (condition 4 in Table 1).

As shown in Figure 6c, the results showed the same tendency as those for PASS under condition 2 in Section 3.3.1, but the value of C.I. with 10^−2^ dilution was lower (see Table S1 for *p*-values). It is suggested that diacetyl is more likely to volatilize when diluted with ultrapure water than when diluted with wash buffer solution. The gelatin-based wash buffer solution used in conditions 2 and 3 has been reported to have a moisturizing effect [31], and it is considered that evaporation is slightly suppressed compared with that with ultrapure water. As a result, the concentration of diacetyl in air under condition 4 with ultrapure water was slightly higher than that in condition 2, and the number of animals that escaped from ponds filled with diacetyl solution with 10^−2^ dilution increased. 

#### 3.3.4. Effect of Size of the Plate on Performance of the PASS

To evaluate the effect of size of the plate on performance of the PASS, we employed circular plates with a diameter of 6 cm instead of circular plates with a diameter of 10 cm in condition 2. We performed three independent assays based on the PASS method under this condition (condition 5 in Table 1). 

As shown in Figure 6d, the results showed the same tendency as those of the PASS under condition 4 in Section 3.3.3 that used the same solvent (ultrapure water), but the dilution at which the value of C.I. changed to a positive value from a negative one was 10^−3^ (see Table S1 for *p*-values). The number of animals trapped in the control pond, C_1_, was extremely low compared with those at other concentrations (Figure S3e). In fact, some animals were paralyzed by diacetyl and remained in the area between the start position and the control pond (data not shown) by the time other animals reached the pond. This suggests that a smaller size of the PASS plate is associated with a higher concentration of diacetyl in air of the plate. Considering the result with 10-cm plates in condition 4, diacetyl solution at 10^−3^ dilution in a 6-cm plate may correspond to that at 10^−2^ dilution in a 10-cm plate. PASS is possible with a 6-cm plate, but higher resolution may be obtained with a 10-cm plate. 

#### 3.3.5. Relationship between the Number of Assay Iterations and Reliability of the PASS Results

To determine the appropriate number of trials of the PASS, we compared the results of three and six independent assays under the same condition (condition 3 in Table 1). The results of the PASS did not differ among the three assays (N = 3) in Figure 6b and six assays (N = 6) in Figure S2a (see Table S1 for *p*-values). Furthermore, we compared the results of three and six independent assays under the same condition (condition 4 in Table 1). The results of the PASS did not differ among the three assays (N = 3) in Figure 6c and the six assays (N = 6) in Figure S2b (see Table S1 for *p*-values). From these results, it was confirmed that PASS is a method that shows high reproducibility, and reliable data can be obtained by three independent assays. 

### 3.4. Detection of Chemotaxis of C. elegans to a Concentration Gradient of NaCl

To evaluate the effectiveness of the PASS as a method for detecting taste chemotaxis, we selected 100 mM NaCl as a single taste chemical to which the preference of *C. elegans* has been well investigated. We performed the conventional chemotaxis assay and the PASS to detect the response of *C. elegans* to 100 mM NaCl. 

**Figure 7:**
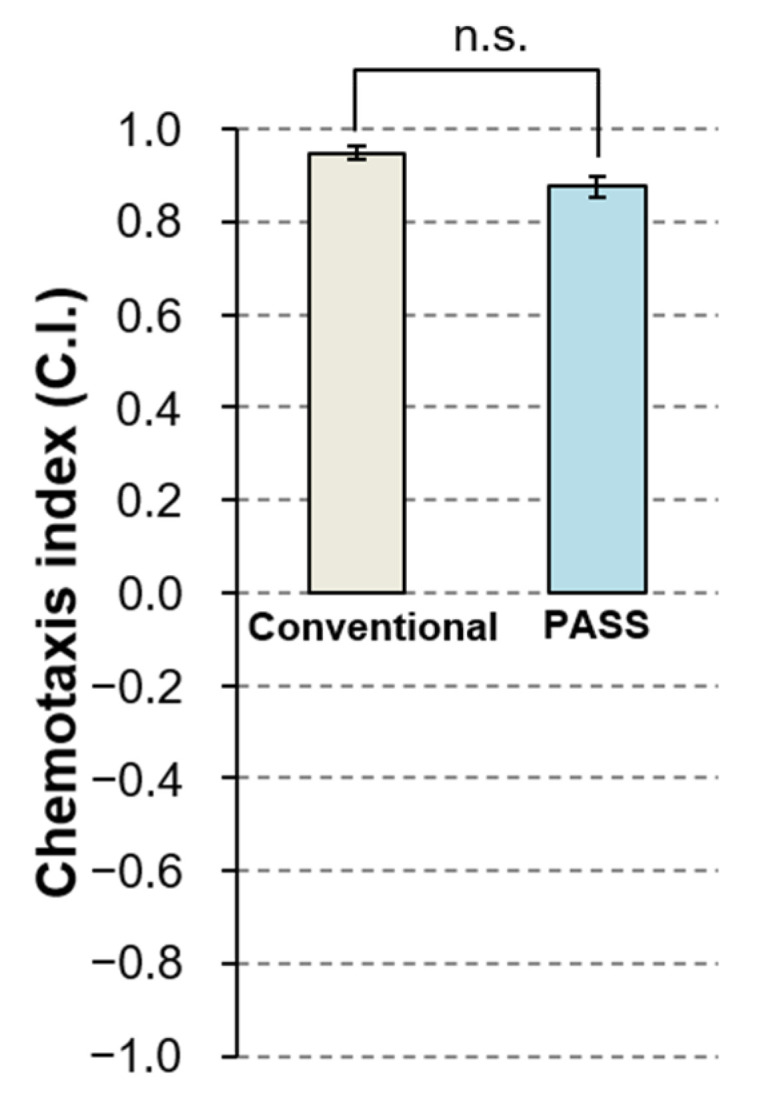
Comparison of the results of the conventional assay and PASS to evaluate chemotaxis of *C. elegans* to a concentration gradient of 100 mM NaCl. Bar and error bar represent the mean and SEM of chemotaxis index (C.I.) of three independent assays under the same condition. C.I. = (the number of animals on the test side−the number of animals on the control side)/(the number of animals on the test side + the number of animals on the control side). Left bar (light charcoal) indicates the result of the conventional assay and the right one (light-blue) indicates the result of PASS. All data were analyzed by Student’s *t*-test at the *p* < 0.05 significance level. n.s. indicates there was no significant difference.

As shown in Figure 7, the C.I. values of both the conventional method and the PASS method were positive, meaning that the animals preferred 100 mM NaCl. The significance of differences between the conventional method and PASS method was examined using Student’s *t*-test because the homoscedasticity was confirmed by F-test (*p* = 0.59). The findings showed no significant difference between the results of the two methods. This confirmed that the taste chemotaxis assay can be performed by the PASS method instead of the conventional method. 

## 4. Discussion

### 4.1. PASS Method without Anesthesia Improves Experimental Efficiency and Accuracy 

We proposed a novel PASS method that can replace the conventional method of chemotaxis assay. The PASS has the advantage that the animals that are attracted to and repelled by the substances cannot return to the solid medium once they fall into the pond. Therefore, the number of animals trapped in a pond in this manner can be accurately evaluated regardless of the elapsed time. Additionally, the number of animals trapped in a pond may increase over time, but cannot decrease. The response evaluation time can be set from a few minutes to a few hours after the start of the test, with consideration of the time it takes for the test and control liquids to evaporate. Furthermore, for unknown reasons, almost all of the released animals fell into one of the ponds, with no individuals escaping to the walls or lids of the plates. Therefore, the burden on the experimenter is dramatically improved.

The details of the numbers of animals that moved to each area in the conventional assay of diacetyl and those trapped in each pond in the PASS are shown in Figure S3a–e. Interestingly, only in the condition in which ponds on the test side (T_1_ and T_2_) and those on the control side (C_1_ and C_2_) were all filled with standard (control) solution did numerous animals choose neither of the ponds (control in Figure S3b–e), and tended to stay around the center of the PASS plate (data not shown). Considering the result that animals were trapped in either of the ponds in the diacetyl assay, it can be stated that animals certainly chose a favorite pond and escaped from the disliked one(s).

Additionally, the anesthesia used to keep animals in the attracted area in conventional chemotaxis assays was no longer required, allowing a pure evaluation of the attractant or repellent response to specific substances and temperatures. 

The PASS itself can be greatly streamlined because the preparation can be completed simply by providing a pond and filling the liquid. In addition, the number of animals in the pond can be measured by imaging or observing the inside of the pond instead of analyzing the entire solid medium, as is the case in the conventional assay, and thus the area to be observed is limited. As such, the response can be easily evaluated and the efficiency can be greatly improved. In the future, it is desirable to perform assays with fewer animals and to develop an efficient method that does not require an assay plate while utilizing the principle of PASS. In particular, introducing microfluidic screenchip technology [31,32] may be a powerful tool for this purpose.

### 4.2. Advance of Assay Method Leads to a Correct Understanding of the Sensory Ability of Living Organisms

We proposed a pond assay for the sensory systems of *C. elegans* as an improved method of evaluating behavioral response and a certain experimenter was performed assays in the same experimental environment. The results of the present study showed that there were no significant experimental errors between the results of almost all assays under the same experimental conditions. In this situation, only cases where a large difference between assays was observed can be regarded as being due to differences among the *C. elegans* individuals themselves. Thus, this novel assay, PASS, for the first time enables clarification of the odor sensory performance of *C. elegans*, which cannot be evaluated correctly by the conventional method. 

Although we here demonstrated the effectiveness of PASS and the olfactory performance of *C. elegans* mainly in chemotaxis assays on diacetyl, which is an odorant, it is necessary to widely re-evaluate the other sensory abilities of *C. elegans* based on PASS in future work. In particular, there is a need for chemotaxis assays to investigate the response to substances containing multiple components, rather than single-component substances. PASS is like a method that digitizes the conventional analog inspection via a very simple mechanism; in the near future, it might provide knowledge of the abilities of sensory systems in *C. elegans* that remain incompletely understood.

## 5. Conclusions

We proposed a pond assay for the sensory systems (PASS) of *C. elegans* as a novel method of behavioral analysis. In PASS, at least a recess is formed on solid agar medium and filled with liquid containing the test subject(s), or at least a recess is formed at the center of a chemical or thermal gradient and filled with liquid. Then, the response of *C. elegans* to the test subject(s) in/around the pond(s) is examined. We reported the effectiveness of PASS as a novel assay for evaluating odorant and taste responses. The results of PASS of diacetyl showed that the response to extremely low concentrations such as 10^−6^ dilution can be clearly detected by using the simple procedure. By using the PASS method, the olfactory system of *C. elegans* that might accurately sense odors at far lower concentrations than previously known will be investigated in the near future. Furthermore, this method can be applied to any technology and it can be utilized in various industrial fields, such as medicine and medical care [33,34,35,36], life sciences, veterinary medicine, agriculture and forestry, food, quantum science, and environmental fields. 

## 6. Patents

The National Institutes for Quantum Science and Technology (QST) to which the authors belong has applied for a PCT international patent related to this technology (PCT/JP2020/017674).

## Figures and Tables

**Table 1:** Conditions for PASS of chemotaxis of *C. elegans* to a graded series of concentrations of diacetyl.

Condition	Method	Plate Size	Volume of Agar	Number of Ponds		Solvent	Control Solution	Volume of Liquid	Figure
				Test	Control				
1	Conventional	⌀10 cm	10 mL	- (2 spots)	- (2 spots)	Wash buffer	Wash buffer	1 µL	5, S3a
2	PASS	⌀10 cm	10 mL	2	2	Wash buffer	Wash buffer	35 µL	6a, S3b
3	PASS	⌀10 cm	20 mL	2	2	Wash buffer	Wash buffer	80 µL	6b, S2a, S3c
4	PASS	⌀10 cm	10 mL	2	2	Ultrapure water	Ultrapure water	35 µL	6c, S2b, S3d
5	PASS	⌀6 cm	3 mL	1	1	Ultrapure water	Ultrapure water	25 µL	6d, S3e

## Data Availability

Not applicable.

## Supplementary Materials

The following are available online at https://www.mdpi.com/article/10.3390/biology11020335/s1, Figure S1: Schematic of conventional plates described in Section 2.2; Figure S2: Results of PASS to evaluate chemotaxis of *C. elegans* to a graded series of concentration of diacetyl; Figure S3: Numbers of *C. elegans* individuals trapped in test side/ponds and control side/ponds in chemotaxis assays of diacetyl; Table S1. The *p*-values from comparisons between the control group and each dilution group for the results shown in Figure 5, Figure 6 and Figure S2; Software S1: The worm counting support software version 1.0.
