# Supplementary material for: Pond Assay for the Sensory Systems of Caenorhabditis elegans: A Novel Anesthesia-Free Method Enabling Detection of Responses to Extremely Low Chemical Concentrations
Source: Biology (Basel). 2022 Feb 21;11(2):335. doi: 10.3390/biology11020335 (PMC8868598; doi:10.3390/biology11020335)
Supplement: Supplementary file 1 [file biology-11-00335-s001.zip › Supplementary Figures and Tables.pdf]

biology

MDPI

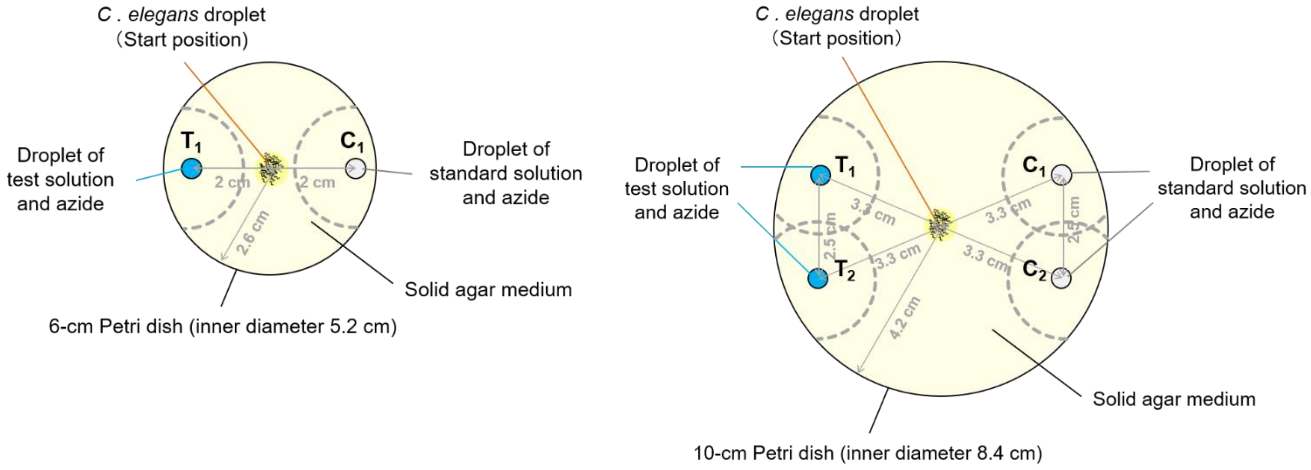

(**a**) (**b**)

Figure S1. Schematic of conventional plates described in section 2.2. (**a**) A ⌀6-cm plate in which test solution and standard (control) solution were spotted on T_1 and C_1, respectively. (**b**) A ⌀10-cm plate in which test solution was spotted on T_1 and T_2 and standard solution was spotted on C_1 and C_2.

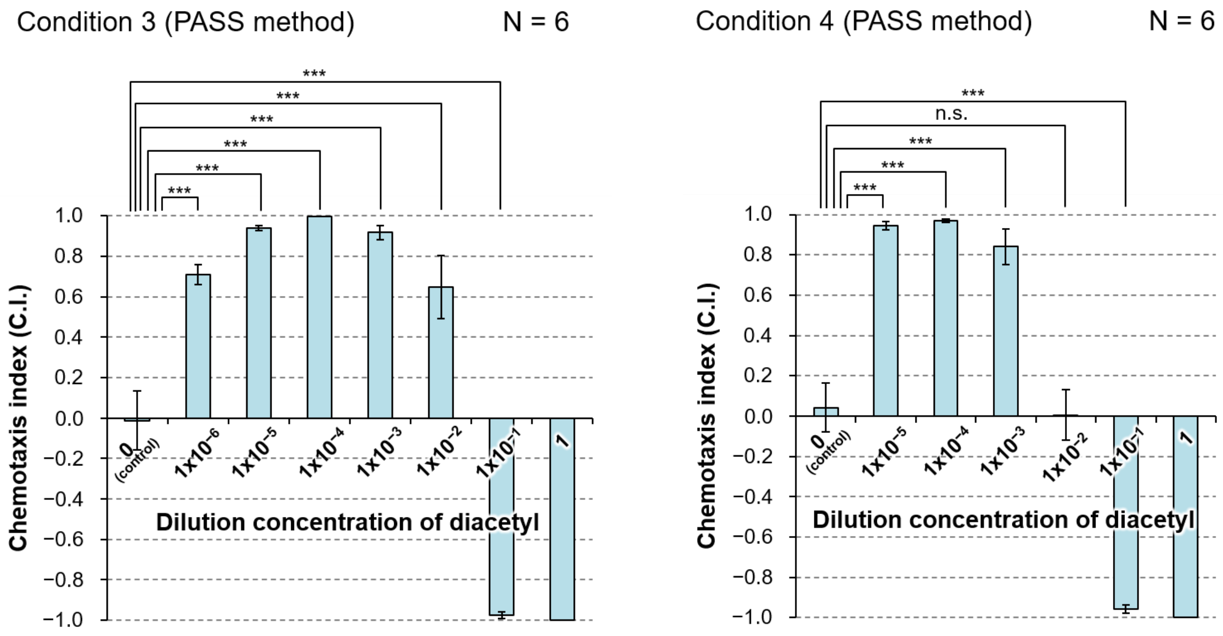

(a) (b)

Figure S2. Result of PASS to evaluate chemotaxis of *C. elegans* to a graded series of concentrations of diacetyl. (**a**) Result of PASS under condition 3 in Table 1. (**b**) Result of PASS under condition 4 in Table 1. The maximum concentration of diacetyl was 1 (undiluted solution) and the minimum diluted concentration was 1×10^{-6} for **a** or 1×10^{-5} for **b**. Light-blue bar and error bar represent the mean and SEM of chemotaxis index (C.I.) of six independent assays under the same condition. All data were analyzed by one-way ANOVA at $p < 0.05$, $p < 0.01$, and $p < 0.001$ (***) significance levels by comparison to the control group. Note that the data of the undiluted solution with no variance were excluded from the analysis. n.s., not significant; N, the number of assays. The details of the p-values are shown in Table S1.

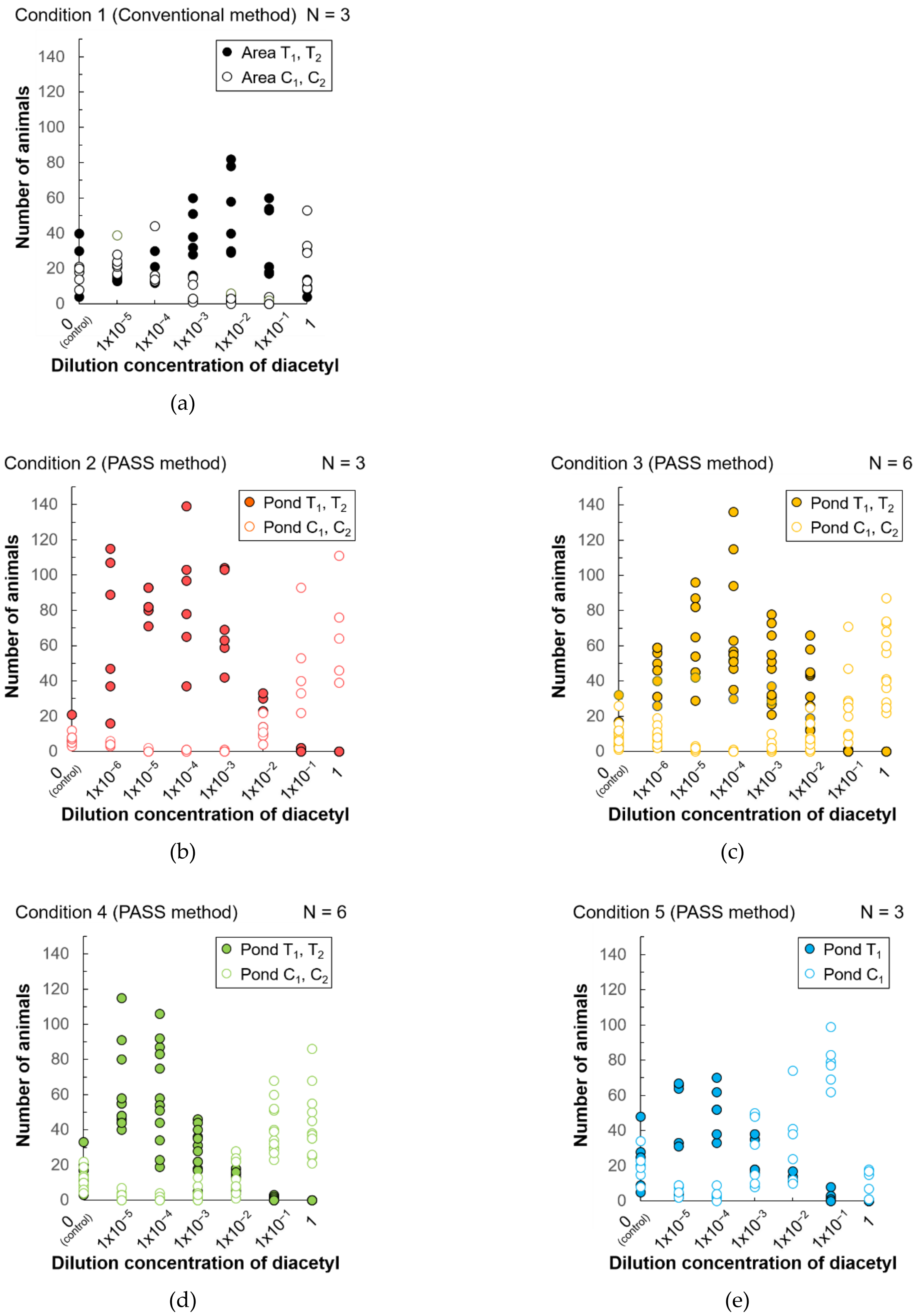

Figure S3. Number of *C. elegans* individuals trapped in the test area/ponds and control area/ponds in chemotaxis assays of diacetyl. (**a**) Result of conventional chemotaxis assay under condition 1 corresponding to Figure 5. (**b**) Result of PASS under condition 2 corresponding to Figure 6**a**. (**c**) Result of PASS under condition 3 corresponding to Figures 6**b** and S2**a**. (**d**) Result of PASS under condition 4 corresponding to Figures 6**c** and S2**b**. (**e**) Result of PASS under condition 5 corresponding to Figure 6**d**. Closed circle represents the number of animals trapped in the area/ponds of the test side, T_1 and T_2, and open circle represents the number of animals trapped in the area/ponds of the control side, C_1 and C_2 at each of three or six independent assays. N, the number of assays.

Table S1. The *p*-values from comparisons between the control group and each dilution group for the results shown in Figures 5, 6, and S2.

Figure \ Dilution		$1x10^{-6}$	$1x10^{-5}$	$1x10^{-4}$	$1x10^{-3}$	$1x10^{-2}$	$1x10^{-1}$	1
Figure 5			0.1933	0.7336	0.0051	0.0006	0.0004	0.1028
Figure 6	**a**	< 0.000001	< 0.0000001	< 0.0000001	< 0.0000001	0.0063	< 0.0000001	Not applicable
	**b**	0.0004	< 0.0001	< 0.0001	< 0.0001	0.0003	< 0.0001	Not applicable
	**c**		< 0.0001	< 0.0001	0.0002	0.8512	< 0.0001	Not applicable
	**d**		0.0031	0.0012	0.6585	0.1767	0.0010	Not applicable
Figure S2	**a**	< 0.000001	< 0.00000001	< 0.000000001	< 0.00000001	< 0.00001	< 0.00000001	Not applicable
	**b**		< 0.00000001	< 0.00000001	< 0.0000001	0.7515	< 0.00000001	Not applicable
